# Elaboration and Characterization of WMoTaNb High Entropy Alloy Prepared by Powder Metallurgy Processes

**DOI:** 10.3390/ma15155416

**Published:** 2022-08-05

**Authors:** Mathias Moser, Sarah Dine, Dominique Vrel, Loïc Perrière, Rémy Pirès-Brazuna, Hervé Couque, Frédéric Bernard

**Affiliations:** 1 Nexter Munitions, 7 Route de Guerry, 18000 Bourges, France; 2 Université de Bourgogne Franche-Comté, ICB–UMR 6303 CNRS, 21000 Dijon, France; 3 Université Paris 13, LSPM–UPR 3407CNRS, 93430 Villetaneuse, France; 4 Université Paris Est Creteil, CNRS, ICMPE, UMR7182, 94320 Thiais, France

**Keywords:** high entropy alloy, self-propagating high-temperature synthesis, spark plasma sintering

## Abstract

This work concerns the sintering of tungsten-based (i.e WMoTaNb) high entropy alloy (HEA) powders using the spark plasma sintering (SPS) technique and their mechanical properties. The synthesis was performed by a self-propagating high-temperature synthesis (SHS) type reaction in which the mixture of metallic oxides (WO_3_, MoO_3_ …) is reduced by magnesium. For this, a specific reactor has been developed. Different conditions including the addition of a moderator were tested. These powders are then densified by SPS technology which allows for keeping the initial microstructure of the powder. The optimization of sintering conditions was performed with the objective to control simultaneously the chemical composition, the grain growth and the densification stages.

## 1. Introduction

High entropy alloys are a recent research field as the first publications defining them date from 2004 [1,2,3,4,5,6]. Contrarily to traditional alloys based on a principal element containing minor elements that allow for adjusting properties (mechanical, corrosion…), high entropy alloys contain several main elements and can be defined as baseless alloys, i.e., they are located in the central region of their phase diagrams. Senkov et al. [7] have shown that many definitions have been given to these alloys according to the number of elements, the entropy configuration, the composition or the number of phases. Some of these definitions are controversial, which is why the term complex concentrated alloy is now preferred to describe both the first generation of HEAs (three to five elements, 5 to 35% at. and a single phase) and the multi-principal element alloys (alloys without principal elements, often called HEAs within first publications).

Many of these alloys are based on refractory metals for their hardness, strength, and stability, especially at high temperatures [8,9]. The WMoTaNb alloys, sometimes associated with vanadium, were the first refractory alloys studied [10]. However, the weak point of such refractory alloys having generally a bcc structure is their low ductility at low temperatures [11]. The synthesis of these refractory alloys is generally made by foundry processes, which are very costly due to the necessity to start from pure metals and consequently the energy required to reach the liquid state. Therefore, the powder metallurgy processes have been selected for this study. There are three types of methods for the preparation of metal powders: chemical methods, physical methods, and mechanical methods. The physical methods are all the techniques of atomization by fluid (liquid or gas) [12]. The principle is to spray a molten metal with a fluid to form droplets that solidify and form spherical particles by a convective growing their cooling. The mechanical method consists of mixing the different materials during a very high-energy grinding [13,14,15,16]. This process allows the synthesis of phases in equilibrium but also out of equilibrium. Apart from the synthesis of powders, high-energy milling has another advantage. The generation of structural defects during the succession of fracture/welding processes permits the storage of energy which will permit the reduction of activation energy of the powder densification allowing for sintering at a lower temperature [17].

Finally, the last method studied in this work concerns the chemical routes which consist of a reduction of metallic oxide powders by a reducing agent, the by-products being then eliminated by stages of purification (leaching, washing). The synthesis of the powders was done by the self-propagating high-temperature synthesis (SHS) method already studied for the synthesis of MoWNb by Dine et al. [18]. A mixture of oxide powders is reduced by an exothermic redox reaction with magnesium. The high entropy alloy is then obtained directly in the powder bed after the removal of the magnesium oxide by lixiviation. Several parameters can be modified to change the synthesis conditions, in particular the diluent which allows for modification of the synthesis temperature. By its principle, this technique is interesting for the preparation of highly refractory alloys compared to the classical metallurgy processes since it does not require a passage to the liquid state. This study continues Sarah Dine’s work [19] on the preparation of tungsten powders by SHS by using it for the synthesis of high-entropy alloys.

Once the powder is prepared, several techniques can be used to densify these powders such as spark plasma sintering (SPS), hot compaction (HP) or selective laser melting (SLM). Spark plasma sintering allowed powder densification by solid-state diffusion with reduced grain growth using fast heating by the Joule effect and the application of a uniaxial load [20,21,22,23,24,25]. The passage by the liquid state is not necessary, which makes it possible to control the microstructure of the part and, thus its properties. This technique is used on many materials, not only metals [26,27,28,29]. One of the major differences from other techniques is the efficiency of the heating. Indeed, the high heating rates (up to 600 °C/min) allow for limiting the growth of grains during sintering [30]. Moreover, starting from mechanically activated powders, dense and nanostructured compounds may be obtained. Indeed, because of a repeated fracture/welding process, both the grain size is reduced, and the defect rates are increasingly changing the activation energy of the sintering. Consequently, the sintering temperature is decreased allowing to maintain the fine microstructure of initial powders leading to improved properties [31].

The advantage of SHS is the use of oxides, less expensive than pure metal powders, which are particularly useful for industrial use. The objective of this study is to sinter by SPS the high entropy alloy powder WMoTaNb prepared by a large volume SHS reactor. The characterization of the samples after sintering will allow for evaluation of the interest in this way of elaboration at the industrial level.

## 2. Materials and Methods

**Figure 1:**
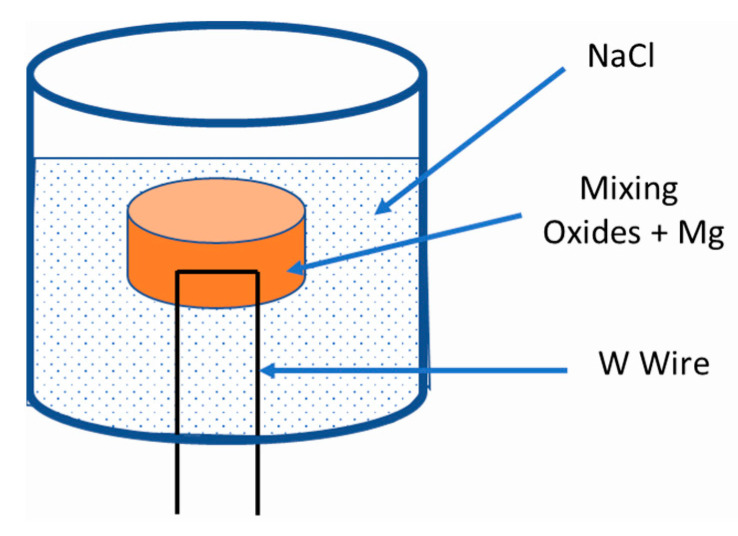
Scheme of the SHS reactor.

WO_3_, Nb_2_O_5_, Ta_2_O_3_ and MoO_3_ powders (99% purity delivered by Alfa Aesar) with pure magnesium powder (99% purity Alfa Aesar <100 µm) are mixed for 2 h in a Formula 8 L V-shaped mixer. Then, the powder mixture is placed in the SHS reactor under an air atmosphere, surrounded by NaCl to avoid contact between the reacting mixture and the reactor walls (Figure 1).

NaCl also plays the role of a moderator of the exothermic reaction. The amount of NaCl allows the control of the maximum temperature reaction. Once the reactor is closed, the reduction of oxide mixtures by the magnesium is ignited by a heated tungsten wire located inside the pellet. The resulting powder is then acid-treated using 2N hydrochloric acid under stirring and heating at 80 °C to dissolve simultaneously NaCl and MgO compounds formed during the reaction. To remove the remaining HCl, the powder is washed with deionized water, then dried in an oven.

To identify some reactions that may occur during sintering, thermogravimetry (TGA) and differential scanning calorimetry (DSC) analyses were conducted on the SHS powders. The analyses were performed under pure Ar and Ar/H_2_(2%) at a flow rate of 50 mL per minute. The interest in this reducing atmosphere is that this latter is close to that maintained in SPS equipment. The powder was heated to 1300 °C at a rate of 20 °C per minute.

For sintering, the powder is placed inside a graphite mold of 30 mm inner diameter and 50 mm in height. To ensure contact between punches, die and the powder, graphite sheets are placed between the graphite tools and the powder. To limit thermal gradients inside the sample during the sintering, graphite felt is placed around the die. Samples of 30 mm diameter and 7 mm high are sintered in an FCT HPD-125 spark plasma sintering machine. The sintering is performed in a vacuum with heating insured by applying a high current. The heating rate is 50 °C/min from 400 °C, at which temperature the pyrometer allows to measure and control the temperature. The plateau temperature is fixed at 1600 °C, with a holding time of 10 min. The uniaxial stress is applied gradually from 700 °C to 1600 °C up to 35 MPa. After sintering, the graphite foil on the sample surface is removed by a sanding operation.

The elementary chemical analysis was performed on the XRF Bruker Tiger S8 device. The microstructure analysis of the material was carried out using a Scanning Electron Microscope Zeiss Merlin with FEG and EDX X-max Oxford Instruments detector for chemical analysis. For X-ray diffraction analysis a Panalytical X’Pert Pro with a PixCel fast detector and Co anticathode (wavelength 0.1789 nm) was used. The specific surface of the powders was determined by nitrogen adsorption sorptometry using a Tristar II Micromeritics coupled to a Smart VacPrep Micromeritics. The test took place at −196 °C preceded by a degassing at 150 °C for 10 h. The reactivity of the powder was studied by thermogravimetric analysis with a STA449C Jupiter Netzch thermo-balance. The analyses were performed from 20 to 1300 °C under two different atmospheres, under argon and H_2_/Ar mixture at a flow rate of 50 mL/min in both cases. To evaluate mechanical properties like the yield strength and the ductility, compression tests were carried out at a rate of 0.0236 mm/s on cylinders of diameter 9 mm and height 5 mm extracted from sintered samples.

## 3. Results

### 3.1. Powder Characterization

**Figure 2:**
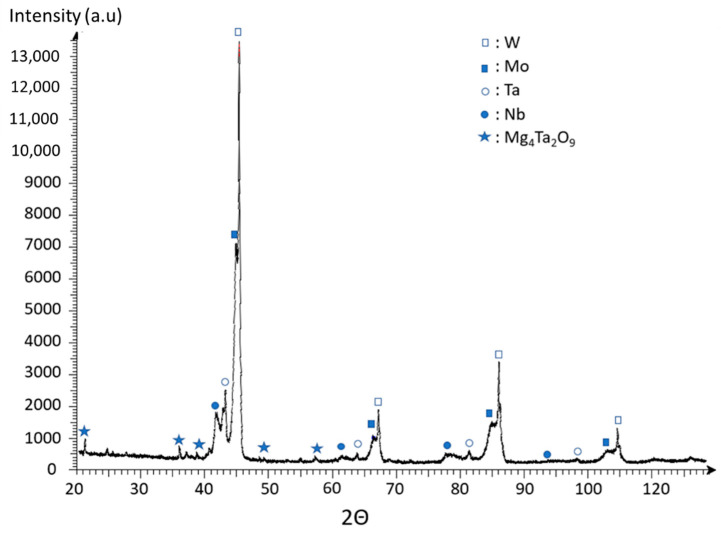
X-ray diffractogram of WmoTaNb powder prepared by SHS.

To verify the powder composition after preparation by SHS an elementary chemical analysis made by XRF (X-ray Fluorescence) has been realized. Results are given in Table 1, showing that the chemical composition obtained after the reduction reaction is close to that expected. Variation may be attributed to the presence of different oxides such as the Mg_4_Ta_2_O_3_ confirmed by the X-ray diffraction (XRD) analysis presented in Figure 2. Moreover, helium pycnometer analyses give much lower density values (8.83 g/cm^3^) than those theoretically expected (14 g/cm^3^), which confirmed once again the presence of the various elements in the form of oxides and not in their metallic form.

The XRD patterns performed of powders issued from the reduction stage (Figure 2) reveal the presence of different metallic phases showing the effectiveness of the reduction by Mg. However, the phases detected are those of the four pure elements W, Mo, Ta, Nb. Unfortunately, such a reaction does not allow the production of a unique HEA (WmoTaNb) phase.

**Figure 3:**
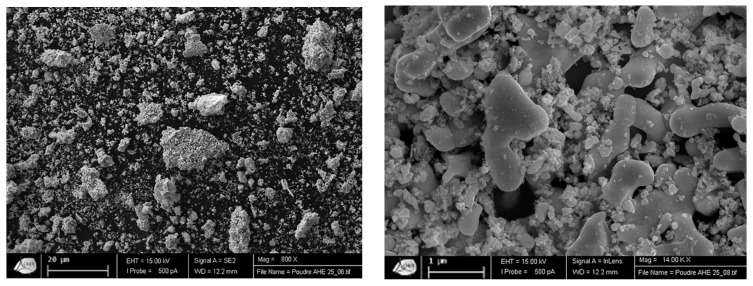
Scanning electron microscope observation of WmoTaNb powder in backscattered electrons.

In fact, an intimate mixture composed of pure metallic elements seems to be obtained, which is confirmed by SEM observation of the powder (Figure 3). This picture exhibits two populations of particles: larger particles of few microns (up to 20µm) and smaller particles with a nanometer size. This site has been confirmed by the determination of the specific surface area, determined by the BET method. Indeed, the calculated surface area is 16.17 ± 0.16 m^2^/g, corresponding to an average particle size of 20 nanometers.

Moreover, SEM observations combined with EDX analysis also indicate that the powder grains are very inhomogeneous in terms of chemical composition, which confirmed that the reduction and purification of the powder are not complete.

**Figure 4:**
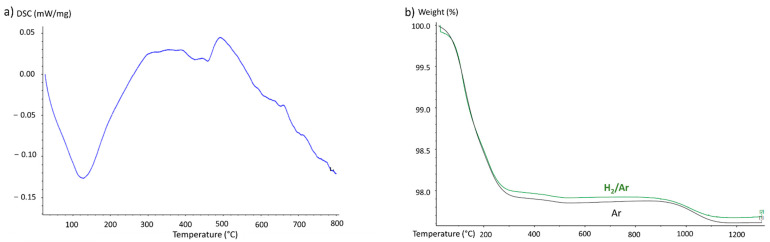
(**a**) DSC analysis of WmoTaNb powder under Ar. (**b**) Thermogravimetric analysis of the milled powder under Ar and Ar/H_2_.

The impurities and fine size of some of the powder particles highlighted by the analyses may both induce a significant reactivity of the powder but also reduce the compressibility of the powder. Indeed, the density of the powder after cold pre-compaction does not exceed 25% of the theoretical density after sintering and is therefore far from being satisfactory for densification by SPS. To increase the powder compressibility, it was decided to mill the powder under argon. The aim of this milling is to break the existing agglomerates and create new ones for which the interparticle interactions are less strong allowing an easier reorganization of the powder for greater compactness. Moreover, the formation of agglomerate will reduce the specific surface areas and thus the reactivity of the powder. For this purpose, the powders were ground for 2 h in a Fritsch PULVERISETTE 4 equipped with hardened steel jars and hardened steel balls. The milling has been realized under argon with a ball-to-powder mass ratio of 2 and a rotation speed of 150 rotations per minute. The thermogravimetric analysis conducted on the powders is shown in Figure 4.

The results highlight a significant mass loss that can be divided according to two temperature ranges. The first one between 20 and 250 °C is the most important and reaches up to 2.2% corresponding to an endothermic reaction on DSC curves. This loss most probably corresponds to the evaporation of the water contained in the sample. The second weight loss begins near 900 °C, corresponding to the volatilization of MoO_3_. The DSC curve shows that an endothermic reaction begins near 500 °C, but without the mass loss shown on the TG curve, this can be explained by the melting of oxides.

The presence of oxides would justify sintering under hydrogen, but the thermogravimetric analyses presented above show that there is no deoxidation during heating under hydrogen. Moreover, due to the large affinity of Ta and Nb for hydrogen, there is a risk of forming hydrides which will be trapped in the sample after densification. Moreover, an analysis under H_2_/Ar atmosphere has been realized to test if a reduction of metallic oxides by hydrogen is efficient but no difference has been noticed between these two atmospheres.

Consequently, different heat treatments have been tested under vacuum, under air at 100 °C and at 120 °C. The results were very varied:

- Under the vacuum at 120 °C, the powder mixture reacts with the aluminum crucible.
- Under air at 100 °C, powder projections were observed.
- Under air at 120 °C, an exothermic reaction took place, leading to the combustion of the plastic container and a large part of the powder, the rest being projected on the walls of the oven.

Finally, a fourth heat treatment under argon in a tubular oven with a temperature rise of 50 °C·min^−1^ followed by a 30 min dwell at 500 °C. At the exit of the furnace after cooling, the formation of a yellow oxide layer (characteristic color of W oxides) on the surface was highlighted. When the volume is increased, the oxide layer cracks and the powder is violently oxidized by ignition. XRD analysis of the oxidized powder shows that the only remaining metallic phase is tungsten, other phases being tantalum, tungsten and mixed niobium/tungsten oxides and no crystallized phases containing molybdenum. It is possible that the exothermic reaction caused the sublimation of the volatile oxide MoO_3_, which would explain the change in color of the powder from yellow just after leaving the furnace to grey when all the powder has been oxidized.

The next step will be to perform a “reactive sintering” inside the SPS chamber, i.e., to allow successively the formation of the HEA phase by a solid-state reaction followed by its densification during the application of uniaxial charge.

### 3.2. Reactive Sintering Step

Given the high reactivity of the powder, it was decided to not apply the pressure before 600 °C which corresponds to the end of the solid-state reaction temperature observed previously. This delay to apply the charge allows to enhance the degassing and avoid trapping some gas species inside the sample pores. It can be seen from the curves in Figure 4 that milling has achieved its objective of reducing the reactivity of the powders. Firstly, outgassing is no longer observed below 400 °C, so there is no reaction occurring at this temperature. The sintering temperature is also higher, 1070 °C instead of 1000 °C. Finally, we observe that the sintering stage is more easily stabilized since there is less reaction, fusion or sublimation of oxides.

**Figure 5:**
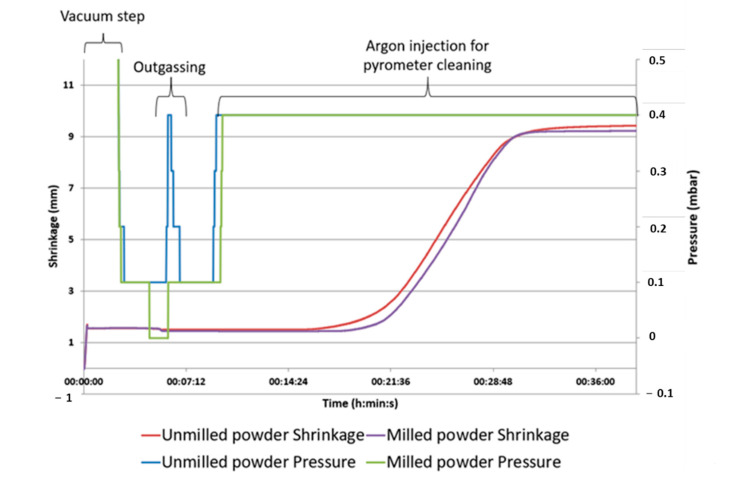
Sintering curves of milled and unmilled WMoTaNb powder.

During a preliminary SPS test, a degassing of the powder was observed below 400 °C (see Figure 5). These outgassing events cause powder splashing inside the SPS as observed previously in the tubular oven. The end-products have lost up to 10% of their initial mass. It is clear that the presence of Mo oxides in the powders which volatilize around 1000 °C also contributes to this mass loss. Unfortunately, outgassing cannot be observed due to the continuous injection of argon in the SPS chamber above 600 °C.

**Figure 6:**
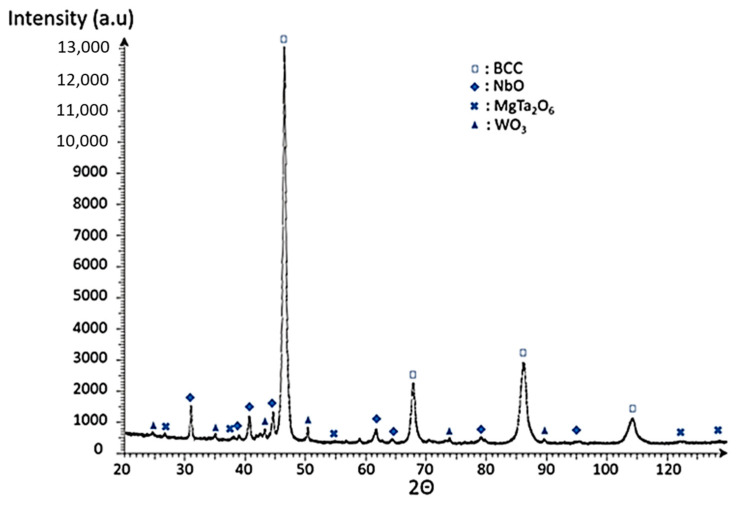
X-ray diffractogram of WMoTaNb sintered by SPS.

The XRD performed on the sintered samples show the presence of many oxides, among them MgO formed during the reaction. However, as the XRD position of MgO is close to those of NbO, it is difficult to exclude this solution (Figure 6). A body-centered cubic (BCC) phase was also formed, which was not present on the diffractogram of the powders before sintering. This confirms that the reactive sintering was effective in forming a HEA phase.

X-ray fluorescence analysis was performed on the sample after sintering (Table 2).

There is a notable gain in the proportion of tungsten, which can be explained by the volatilization of the oxides of the other three elements.

**Figure 7:**
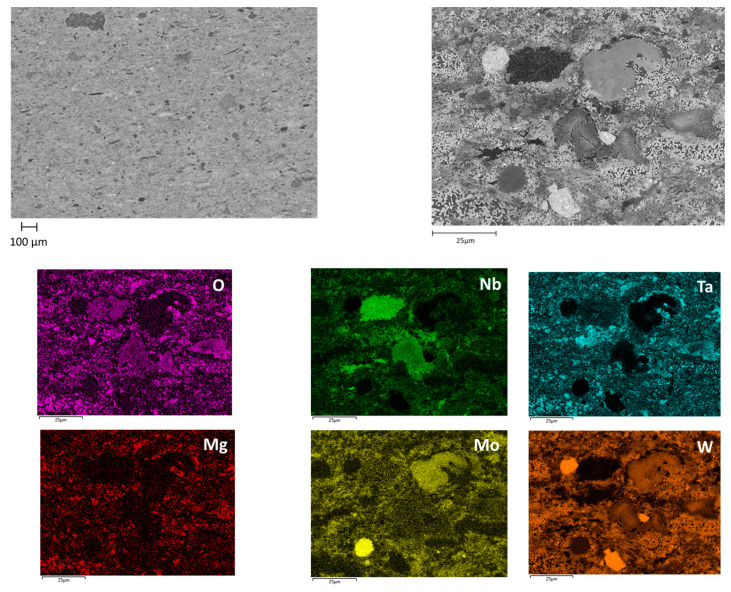
Chemical mapping of the sintered sample at 1600 °C under axial compression of 35 MPa for 10 min.

The SEM and EDX analyses on the sintered samples (Figure 7) exhibit a very heterogeneous chemical composition of the sintered sample: some areas are pure W or pure Mo while others are made of the alloy most likely formed during the SHS. That denotes that little to no long-range diffusion occurred during sintering, meaning that both temperature and time of sintering are not sufficient to improve the homogeneity of the sample.

To control the porosity and the densification of the samples, the volume mass measurements by helium pycnometry were performed on the samples. The density of the sample is 11.36, higher than the powder’s value, confirming the X-ray fluorescence analyses concerning the volatilization of the oxides during the sintering. However, this density is still largely lower than the theoretical value due to the presence of numerous impurities. Thus, it does not seem relevant to compare the measured density with the theoretical value of 15.39 to obtain the relative density and porosity rate.

**Figure 8:**
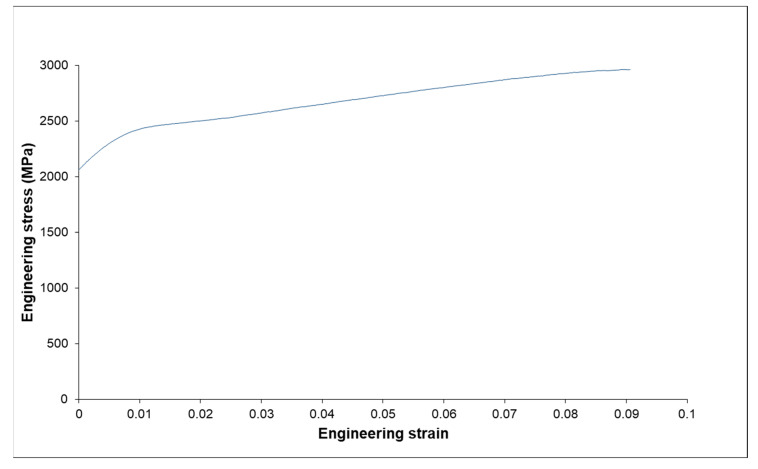
Compression stress strain curve of the sintered sample.

The engineering stress–strain response in compression of the sintered sample is reported in Figure 8. These data are compared in Table 3 to data obtained with samples prepared, respectively, by arc melting and by mechanical activation followed by SPS sintering.

The results clearly demonstrate the advantages of the preparation by powder metallurgy and SPS concerning ductility. The numerous oxides improve the yield strength of the alloy over mechanical activation and vacuum arc melting. As shown by Kim et al. [33], during W sintering with the addition of Y_2_O_3_, the oxide dispersion allows both grain growth limitation and density increase by liquid phase sintering. However, the presence of numerous oxides at the grain boundaries weakens the microstructure and decreases the ductility.

## 4. Conclusions

The high entropy alloy WMoTaNb was synthesized by magnesiothermy. If the X-ray fluorescence analysis showed that the chemical composition was close to the expected one, the presence of numerous impurities, often in the form of oxides, could be identified. Moreover, instead of a single solid solution, the powder obtained is rather in the form of a mixture of metallic phases. The difficulty to obtain a pure and homogeneous powder mixture leads to a large reactivity. Indeed, violent exothermic reactions are observed during heat treatment attempts. The presence of niobium and tantalum, which are particularly sensitive to hydriding, also makes it difficult to treat the powders in a reducing atmosphere to reduce these oxides. Due to the high reactivity of the powders, significant outgassing is observed during the densification of the powders by SPS. This could be reduced by grinding the powders before sintering, the formation of powder agglomerates having reduced the specific surface and thus their reactivity. The sintered sample obtained still presents many oxides and the SEM images show a very heterogeneous microstructure. The X-ray fluorescence shows a higher tungsten content than the powder, which would tend to show that some of the oxides, especially niobium, have been volatilized during sintering. The presence of oxides seems to improve the mechanical strength of the sample, which allows obtaining results superior to those reported in the literature. Even if the presence of oxides weakens the sample, the deformation during the compression tests remains higher than that obtained for a sample prepared by arc melting.

The main issue of this study is the high amount of impurities in the powder prepared from the SHS route. It, therefore, seems necessary to improve this step, either during the synthesis itself or during the purification steps including the application of some thermal treatment. One of the solutions considered is to increase the contact surface between the powder mixture and the NaCl regulator. The aim is to homogenize the reaction temperature and increase the yield of the reaction. If these new conditions are effective, the powder should be less reactive and then it will be easier to perform heat treatments on such powder to remove the last impurities.

## Figures and Tables

**Table 1:** X-ray fluorescence analysis on WMoTaNb powder prepared by SHS.

	%At Experimental	%At Theoretical
**W**	28.0	30.6
**Mo**	20.2	23.1
**Ta**	23.3	21.4
**Nb**	26.4	24.9

**Table 2:** X-ray fluorescence analysis on WMoTaNb sintered by SPS.

	%At Experimental	%At Theoretical
**W**	38.8	30.6
**Mo**	23.6	23.1
**Ta**	21.1	21.4
**Nb**	16.5	24.9

**Table 3:** Yield stress and maximum strain in compression at room temperature for WMoTaNb prepared in this work in comparison with results prepared by other techniques Vacuum Arc melting, mechanical activation and SPS [11,32].

	Vacuum Arc Melting [11]	Mechanical Activation + SPS [32]	SHS + SPS This Work
Yield stress (MPa)	1058	1058	2080 ± 50
Maximum strain (%)	2.6	16.8	9.0 ± 1
